# Titania Nanotubes/Hydroxyapatite Nanocomposites Produced with the Use of the Atomic Layer Deposition Technique: Estimation of Bioactivity and Nanomechanical Properties

**DOI:** 10.3390/nano9010123

**Published:** 2019-01-19

**Authors:** Aleksandra Radtke, Michalina Ehlert, Tomasz Jędrzejewski, Beata Sadowska, Marzena Więckowska-Szakiel, Jani Holopainen, Mikko Ritala, Markku Leskelä, Michał Bartmański, Marek Szkodo, Piotr Piszczek

**Affiliations:** 1 Faculty of Chemistry, Nicolaus Copernicus University in Toruń, Gagarina 7, 87-100 Toruń, Poland; m.ehlert@doktorant.umk.pl (M.E.); piszczek@chem.umk.pl (P.P.); 2 Nano-implant Ltd. Gagarina 5, 87-100 Toruń, Poland; 3 Faculty of Biology and Environmental Protection, Nicolaus Copernicus University in Toruń, Lwowska 1, 87-100 Toruń, Poland; tomaszj@umk.pl; 4 Faculty of Biology and Environmental Protection, University of Lodz, Banacha 12/16, 90-237 Łódź, Poland; beata.sadowska@biol.uni.lodz.pl (B.S.); marzena.wieckowska@biol.uni.lodz.pl (M.W.-S.); 5 Department of Chemistry, University of Helsinki, A.I. Virtasen aukio 1, 00014 Helsinki, Finland; jani.h.holopainen@outlook.com (J.H.); mikko.ritala@helsinki.fi (M.R.); markku.leskela@helsinki.fi (M.L.); 6 Faculty of Mechanical Engineering, Gdańsk University of Technology, Gabriela Narutowicza 11/12, 80-233 Gdańsk, Poland; michal.bartmanski@pg.edu.pl (M.B.); marek.szkodo@pg.edu.pl (M.S.)

**Keywords:** titania, nanotubes, hydroxyapatite, atomic layer deposition, Ti6Al4V

## Abstract

Titanium dioxide nanotubes/hydroxyapatite nanocomposites were produced on a titanium alloy (Ti6Al4V/TNT/HA) and studied as a biocompatible coating for an implant surface modification. As a novel approach for this type of nanocomposite fabrication, the atomic layer deposition (ALD) method with an extremely low number of cycles was used to enrich titania nanotubes (TNT) with a very thin hydroxyapatite coating. X-ray diffraction (XRD) and scanning electron microscopy (SEM) were used for determination of the structure and the surface morphology of the fabricated nanocoatings. The biointegration activity of the layers was estimated based on fibroblasts’ proliferation on the TNT/HA surface. The antibacterial activity was determined by analyzing the ability of the layers to inhibit bacterial colonization and biofilm formation. Mechanical properties of the Ti6Al4V/TNT/HA samples were estimated by measuring the hardness, Young’s module, and susceptibility to scratching. The results revealed that the nanoporous titanium alloy coatings enriched with a very thin hydroxyapatite layer may be a promising way to achieve the desired balance between biofunctional and biomechanical properties of modern implants.

## 1. Introduction

The growing demand for bone implants, which could replace worn bone elements or support their regeneration, is closely related to the active lifestyle of contemporary people, as well as the aging of societies. Numerous groups in the world have carried out intensive studies on the development of new implant technologies and have been searching for novel biomaterials for implant production, with great progress [1]. The aim of these studies is the fabrication of biocompatible materials, which are characterized by non-toxicity, antimicrobial activity, and minimal impact on the immune system. 

Titanium and its alloys are biomaterials widely used in the implants intended for orthopaedics, dentistry, maxillofacial surgery, as well as in laryngology and cardiology [2,3]. This is due to the low toxicity of these materials, their excellent corrosion resistance, high specific surface area, as well as very good mechanical properties. Despite the unique properties of titanium and its alloys, to meet the above-mentioned requirement, associated with high biocompatibility, the implant surfaces must be modified by appropriate mechanical or chemical treatments or by forming composite coatings on them [4,5,6,7]. Moreover, the modified surface of the implant must be resistant to mechanical damage, abrasion, and infections, and it must show biocompatibility [8]. The results of previous works indicate that the formation of titania nanotube (TNT) layers on the implant surface improves its osseointegration while maintaining appropriate mechanical properties [4,5,9,10]. 

Deposition of hydroxyapatite film (HA-Ca_10_(PO_4_)_6_(OH)_2_) on the implant surface seems to be highly beneficial from a biological point of view because HA is the most stable form of calcium phosphate [11,12], which is the natural bone component (bones consist of c.a. 70% hydroxyapatite (HA), 20% type I collagen fibrils, and about 10% water [13,14,15]. Unfortunately, HA coating often negatively affects the mechanical properties of the complete system (metal substrate + HA layer) [16,17]. Studies on the HA nanolayers revealed their tendency to form nano-sized crystals of a high surface area, and thereby result in faster dissolution in body fluids. However, it should be noted that the osteoclastic resorption and the solubility of hydroxyapatite are also sensitive to factors, such as: crystallinity of hydroxyapatite, incorporation of carbonates, and content of other ions [12,18]. A biological HA is usually a calcium deficient and it is always substituted with a carbonate. This decreases the value of the Ca/P atomic ratio, which is equal to 1.67 in the stoichiometric HA [19,20]. HA coatings, in the form of dense films, have shown success in implant fixation (in-vivo), which increases the implant life by increasing the strength and rate of implant integration [21,22,23]. In addition to their bioactive role, hydroxyapatite films deposited on metallic implants can minimize metal ions’ release in the physiological environment of the body [24]. However, HA coatings are brittle and have low strength under wear conditions [25,26]. HA coated implants may also suffer from fatigue damage under cyclic loading in the body [27]. Lynn et al. investigated the effect of HA coating thickness on the fatigue behavior of Ti-6Al-4V [28]. They found that coatings up to 100 um have no effect on the fatigue life of the alloy as compared to the uncoated alloy, but coatings of a thickness of 150 µm considerably reduces the fatigue resistance of Ti-6Al-4V [28]. 

The idea of TNT coating covered with a very thin hydroxyapatite layer seems to be especially promising in improving bone-implant osteointegration processes without affecting the fatigue resistance of Ti6Al4V. An analysis of literature reports indicates that the production of the titanium dioxide layer on the implant surface improves the adhesion and affects the anti-corrosion behavior of the oxide contact layer [2,3,5,6,7,29,30]. However, the increase in thickness of the HA layer leads to a weakening of the mechanical properties of the overall system, which can result in smoothening of the earlier made micro- or nanoscale modifications. Therefore, the deposition rate and coverage, coating morphology, and especially interfacial quality, are of great importance when choosing the right deposition technology for medical applications [19,31,32,33,34]. Hydroxyapatite coatings can be produced by many different techniques, e.g., the hydrothermal method, sol-gel, micro-arc oxidation, electrodeposition, biomimetic deposition, and plasma spraying [31,35,36,37,38,39,40,41]. Atomic layer deposition (ALD) has also been used to synthesize bio-ceramic coatings. ALD is a chemical method, which allows the production of highly conformal and uniform thin films. Due to the alternating precursor pulses with self-limiting reactions, ALD is capable of producing films with atomic layer thickness control and to coat uniformly and conformally complex 3D structures [17,33,42,43,44]. 

The aim of our work was the estimation of the bioactivity and nano-mechanical properties of Ti6Al4V/TNT/HA systems, which may be applied as a potential material for the construction of new generation implants. Nanocomposite TNT/HA coating on the surface of a titanium alloy implant is intended to initiate and accelerate regeneration of the bone tissue and to stimulate osteogenic cells. In our work, we combined three synthetic techniques: (1) Electrochemical oxidation of a Ti6Al4V surface to create a nanotubular titania surface, (2) ALD of a very thin calcium carbonate nanolayer on the titania nanotubes, and (3) wet thermal conversion of CaCO_3_ film to nanocrystalline hydroxyapatite [17,33] to optimize the coating in terms of high biocompatibility and proper mechanical properties. 

## 2. Materials and Methods

### 2.1. Fabrication of Ti6Al4V/TNT/HA Composite Coatings

#### 2.1.1. Anodic Oxidation of Ti6Al4V Substrates and Structural and Morphological Studies of the Ti6Al4V/TNT System

The electrochemical anodic oxidation method was used to fabricate the TNT system on the surface of Ti6Al4V substrates, applying previously described conditions (Ti6Al4V, grade 5 foil, 99.7% purity, 0.20 mm thick, Strem Chemicals, Inc. (Bischheim, France), 7 mm × 70 mm pieces) [6,7]. The anodization time was 20 min, and the process was carried out at potentials: 5 V (TNT5), 10 V (TNT10), 12 V (TNT10), 15 V (TNT15), 18 V (TNT18), and 30 V (TNT30). The structure of the produced TiO_2_ nanotube layers was studied using Raman spectroscopy (RamanMicro 200 PerkinElmer (PerkinElmer Inc., Waltham, MA, USA) (λ = 785 nm)) and diffuse reflectance infrared Fourier transform spectroscopy (DRIFT, Spectrum2000, PerkinElmer Inc., Waltham, MA, USA). The morphology of the coatings was examined using a Quanta scanning electron microscope with field emission (SEM, Quanta 3D FEG, Huston, TX, USA). 

#### 2.1.2. Preparation of Ti6Al4V/TNT/HA Coatings and Their Structural Characterization

**Scheme 1:**
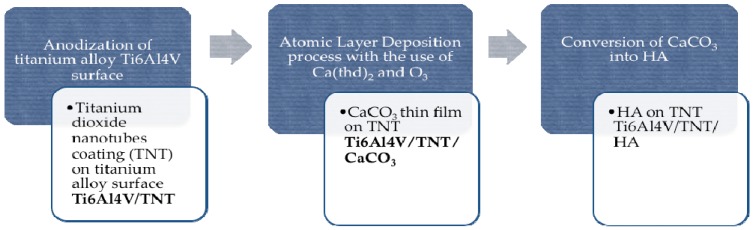
Schematic illustration of the overall Ti6Al4V/TNT/HA synthesis procedure.

Hydroxyapatite was fabricated on the surface of Ti6Al4V/TNT substrates by the two-stage procedure: (1) Deposition of CaCO_3_ thin film using the ALD method and (2) conversion of the CaCO_3_ film into crystalline hydroxyapatite by a reaction with aqueous (NH_4_)_2_HPO_4_ [17,33]. In the first step, the Ti6Al4V/TNT samples were attached onto 5 × 5 cm^2^ silicon with Kapton tape and placed into an F-120 ALD reactor (ASM Microchemistry Ltd., Helsinki, Finland). The CaCO_3_ films were deposited using Ca(thd)_2_ and O_3_ as the precursors at 250 °C; the growth rate for CaCO_3_ was around 0.47–0.48 Å/cycle. Unless otherwise noted, 100 cycles were applied that resulted in an approximately 5 nm CaCO_3_ film thickness. In the next step, the Ti6Al4V/TNT/CaCO_3_ composites were immersed in 0.2 M (NH_4_)_2_HPO_4_ and 1 M NH_3_ aqueous solution, which had been heated to 95 °C. The produced Ti6Al4V/TNT/HA systems were rinsed with deionized water and blown dry. Scheme 1 illustrates the overall Ti6Al4V/TNT/HA synthesis procedure.

The morphology of the fabricated coatings (both stages) was studied using a Hitachi S-4800 field emission scanning electron microscopy, FE-SEM (Hitachi High-Technologies Corporation, Tokyo, Japan). Prior to imaging the samples were sputter coated with 2 nm Au/Pd to enhance image quality. Grazing incidence X-ray diffraction (GIXRD) studies were performed with an X-ray diffractometer PANalytical X’Pert Pro MPD (PANalytical B.V., Almelo, Netherlands) using CuKα radiation and an incident angle of 1°.

### 2.2. Wettability and the Surface Free Energy Measurements

The wettability determination was carried out, by the contact angle measurements, using a goniometer with drop shape analysis software (DSA 10 Krüss GmbH, Hamburg, Germany). The volume of the distilled water drop in the contact angle measurement was 3 μL. To estimate the value of the surface free energy based on mathematical calculations, which were performed using the Owens-Wendt method, the contact angle of diiodomethane (CH_2_I_2_) was measured. The volume of the diiodomethane drop in the contact angle measurement was 4 μL. Each measurement was carried out three times, immediately after deposition of the drop.

### 2.3. Topography and the Mechanical Properties of Studied Systems

#### 2.3.1. Atomic Force Microscopy

To study the surface topography of Ti6Al4V (reference), and the Ti6Al4V/HA and Ti6Al4V/TNT/HA specimens, an atomic force microscope (AFM, NaniteAFM, Nanosurf AG, Liestal, Switzerland) was used. The examinations were performed in the non-contact mode at 55 mN force. The roughness index, Sa, was estimated in the area of 50 × 50 μm.

#### 2.3.2. Mechanical Studies—Nanoindentation and Nano Scratch—Test

Nanoindentation tests were performed with the NanoTest™ Vantage (Micro Materials Ltd., Wrexham, UK) using a Berkovich three-sided pyramidal diamond. Ten independent measurements of indentation were carried out for each sample. The maximum applied force was equal to 10 mN, the loading time was 15 s, the dwell period at maximum load was 10 s, and unloading time was 10 s. The distances between the subsequent indents were 20 μm. Based on the load–penetration curves and the Oliver and Pharr method, the surface hardness (H) and Young’s modulus (E) were calculated using the integrated software. For the estimation of the Young’s modulus, the Poisson’s ratio 0.25 was assumed. The mechanical properties of the nanocomposites, such as the elastic strain to failure of the coating (H/E) and the factor used to describe the resistance of a material to plastic deformation (H^3^/E^2^), were also studied using the nanoindention technique [45,46].

Nano-scratch tests were performed with the NanoTest™ Vantage (Micro Materials Ltd., Wrexham, UK) using a Berkovich three-sided pyramidal diamond. Five independent measurements were made for the Ti6Al4V/HA and Ti6Al4V/TNT/HA specimens. The scratch tests were made by increasing the load from 0 mN to 200 mN at a loading rate of 1.3 mN/s over a distance 500 µm. The adhesion failure of the coating was assessed based on the observation of an abrupt change in frictional force during the test.

### 2.4. Biocompatibility Assays

#### 2.4.1. Cell Culture

Murine fibroblast cell line L929 (American Type Culture Collection, Manassas, VA, USA) were cultured in a complete RPMI 1640 medium containing 2 mM L-glutamine, heat-inactivated 10% fetal bovine serum, 100 µg/mL streptomycin, and 100 IU/mL penicillin (all compounds from Sigma-Aldrich, Darmstadt, Germany). Cultured cells were incubated at 37 °C, 5% CO_2_, and 95% humidity, after which sub-confluent cells (70–80% of surface of flask, covered by cell monolayer, were expanded by cell scraping.

#### 2.4.2. Biointegration Studies of Studied Systems

L929 murine fibroblasts in a volume of 1 mL of culture medium were seeded onto the autoclaved test specimens placed in a 24-well culture plate (Corning, NY, USA) at a density of 1 × 10^4^ cells/well for 72 h or 120 h days. The cells incubated with the test plates were analyzed for the level of cell proliferation as well as the observation of cell morphology.

The effect of TiO_2_ nanocoatings enriched with hydroxyapatite on the L929 cells proliferation (measured after 72 h and 120 h) were studied by the MTT (3-(4,5-dimethylthiazole-2-yl)-2,5-diphenyl tetrazolium bromide; Sigma Aldrich, Darmstadt, Germany) assay using the same method as was reported in [5,6,7]. Briefly, after the respective incubation time, the plates were washed with phosphate buffered saline (PBS), and then the MTT (5 mg/mL; Sigma-Aldrich) solution in an RPMI 1640 culture medium without phenol red (Sigma-Aldrich) was added to each well. After 3 h of incubation, the samples were transferred to new 24-well culture plates and 500 μL of dimethyl sulfoxide (DMSO; 100% *v/v*; Sigma Aldrich) was added to each well. The plates were shaken for 10 min and the absorbance was measured at the wavelength of 570 nm with the subtraction of the 630 nm background, using a microplate reader (Synergy HT; BioTek, Winooski, VT, USA). All measurements were done in duplicate in five independent experiments.

Scanning electron microscopy (SEM; Quanta 3D FEG; Carl Zeiss, Göttingen, Germany) analyses were performed to study the morphology changes of L929 fibroblasts growing on the surface of the studied samples, using the same method as in [5,6,7]. Briefly, after the selected incubation time, the specimens were washed with PBS to remove the non-adherent cells and were fixed in 2.5% *v/v* glutaraldehyde (Sigma Aldrich). Then, the plates were rinsed again with PBS and dehydrated in a graded series of ethanol (50%, 75%, 90%, and 100%). Finally, the samples were dried in vacuum-assisted desiccators overnight and stored at room temperature until the SEM analysis was performed.

#### 2.4.3. Statistical Analysis in the MTT Assay

All values are reported as means ± standard error of the means (SEM) and were analyzed using analysis of variance (ANOVA) followed by a Bonferroni multiple comparisons test with the level of significance set at p < 0.05. Statistical analyses were performed with GraphPad Prism 7.0 (La Jolla, CA, USA).

### 2.5. Antibacterial Test

#### 2.5.1. Bacterial Strains and Biofilm Formation on Studied Systems

*Staphylococcus aureus* ATCC 29213 reference strain (MSSA, methicillin-susceptible *S. aureus*) and *S. aureus* H9 clinical strain (MRSA, methicillin-resistant *S. aureus*) were grown for 24 h at 37 °C on Tryptic Soy Agar—TSA (BTL, Poland) and for the next 18 h on Tryptic Soy Broth—TSB (BTL, Poland) containing 0.25% glucose (TSB/Glu). Finally, microbial suspension at the optical density OD_535_ = 0.6 (nephelometer type Densilameter II, Czech Republic) was prepared. Biomaterial samples were added to 1 mL of bacterial suspensions into the wells of 24-well tissue culture polystyrene plates (Nunc, Denmark) and incubated for 24 h at 37 °C in stable conditions to form a microbial biofilm. *Staphylococcal* suspensions alone (without biomaterial) and TSB/Glu only were used as a positive and negative control, respectively. Two independent experiments with four replicates in each were performed.

#### 2.5.2. The Assessment of *S. aureus* Biofilm on the Studied Systems

A LIVE/DEAD BacLight Bacterial Viability kit (L/D; Molecular Probes, Eugene, OR, USA) was used to assess biofilm formation on the tested biomaterials. First, the biomaterials were dipped in fresh TSB to gently remove bacteria weakly bound with their surface. Then, the pieces of titanium foil were vortexed (3 min) in TSB to reclaim the cells forming the biofilm. The obtained *S. aureus* suspensions or medium (negative control) were added (100 μL) to the wells of 96-well black microplates (Greiner, Germany) in quadruplicate. Bacterial staining was performed as recommended by the manufacturer of the L/D test and finally the fluorescence at ex. 485 nm/em. 530 nm (green fluorescence) and ex. 485 nm/em. 620 nm (red fluorescence) was measured. Based on the relative fluorescence units (RFU), for green fluorescence, a percentage of live bacteria in the biofilm formed on the modified titanium foils tested in comparison to microbial biofilm on a control unmodified biomaterial, considered as 100%, was calculated.

#### 2.5.3. Statistical Analysis

The nonparametric Kruskal–Wallis one-way ANOVA was used to compare differences among the samples from different populations. *P* ≤ 0.05 was considered significant.

## 3. Results

### 3.1. The Structure and Morphology of Ti6Al4V/TNT/HA

**Figure 1:**
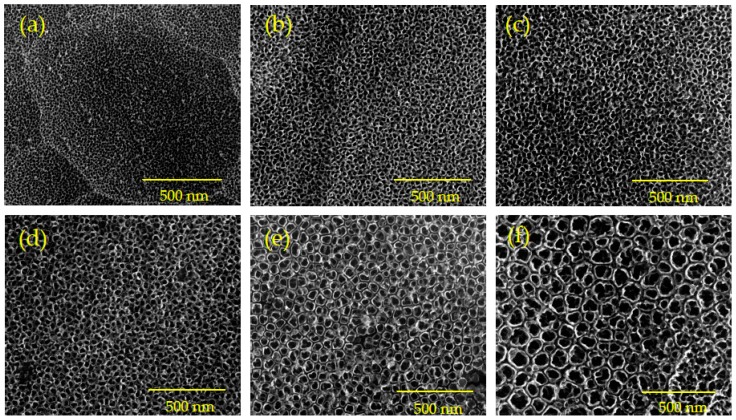
SEM images of TNTs coatings produced on the surface of Ti6Al4V substrates using potentials 5-30V ((**a**) 5 V (Ti6Al4V/TNT5), (**b**) 10 V (Ti6Al4V/TNT10), (**c**) 12 V (Ti6Al4V/TNT12), (**d**) 15 V (Ti6Al4V/TNT15), (**e**) 18 V (Ti6Al4V/TNT18), and (**f**) 30 V (Ti6Al4V/TNT30)).

The anodic oxidation of Ti6Al4V samples in 0.3wt% aqueous HF solution led to uniform nanotubular coatings (TNT), with a tube length of approximately 150–200 nm. The nanotube diameters increased from 18 to 140 nm with increasing potential applied. Surface morphology changes of the produced Ti6Al4V/TNT systems are presented in Figure 1.

**Figure 2:**
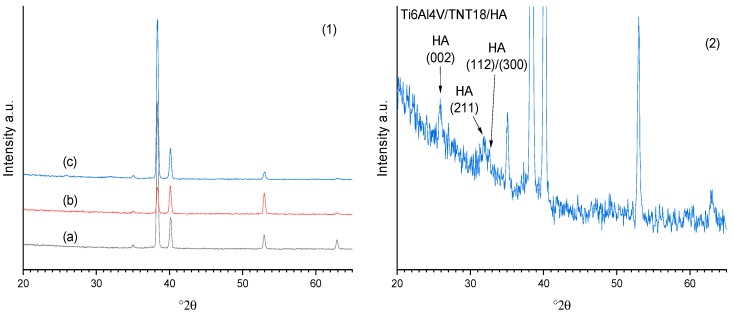
GIXRD of (**1**): (a) Ti6Al4V/TNT18, (b)Ti6Al4V/TNT18/CaCO_3_, (c)Ti6Al4V/TNT18/HA, and (**2**) Ti6Al4V/TNT18/HA. The reflections assigned to HA are marked separately whereas all unmarked peaks were assigned to the substrate.

**Figure 3:**
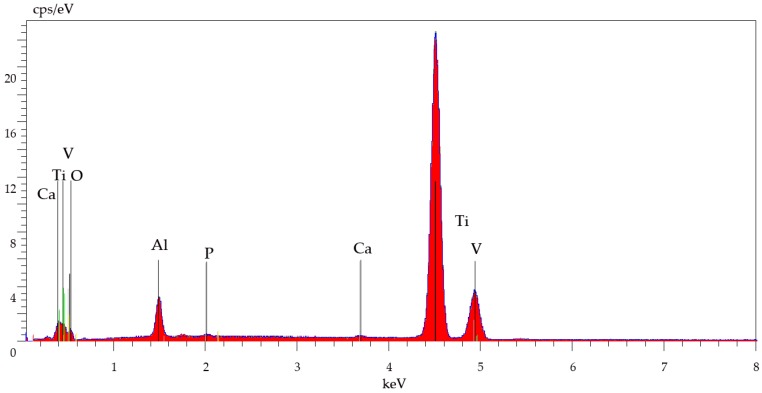
EDS spectrum of Ti6Al4V/TNT5/HA.

The amorphous structure of the produced TNT coatings was confirmed by the Raman and IR DRIFT spectroscopy methods. In the next step of our work, the Ti6Al4V/TNT samples were covered by a CaCO_3_ nanolayer using the ALD technique (100 cycles of Ca(thd)_2_ and O_3_ at 250 °C). Analysis of the grazing incidence X-ray diffraction (GIXRD) patterns of the Ti6Al4V/TNT/CaCO_3_ systems confirmed the amorphous form of the calcium carbonate nanocoatings (Figure 2(1)). In addition, it could be noted that the TNT layer was not crystallized during the ALD process. The CaCO_3_ thin films were converted to hydroxyapatite nanocrystals by immersion into an aqueous (NH_4_)_2_HPO_4_-NH_3_ solution. The structure of the produced HA coatings was confirmed by GIXRD (Figure 2(2)). The HA reflections are clear, but weak, because of the small thickness of the HA layer. The energy dispersive spectrum (EDS) of Ti6Al4V/TNT5/HA, measured in SEM, is shown in Figure 3, revealing the presence of Ca and P on the surface of the sample Ti6Al4V/TNT5/HA. Calculated Ca/P ratios for Ti6Al4V/HA and Ti6Al4V/TNT/HA samples are presented in Table 1.

**Figure 4:**
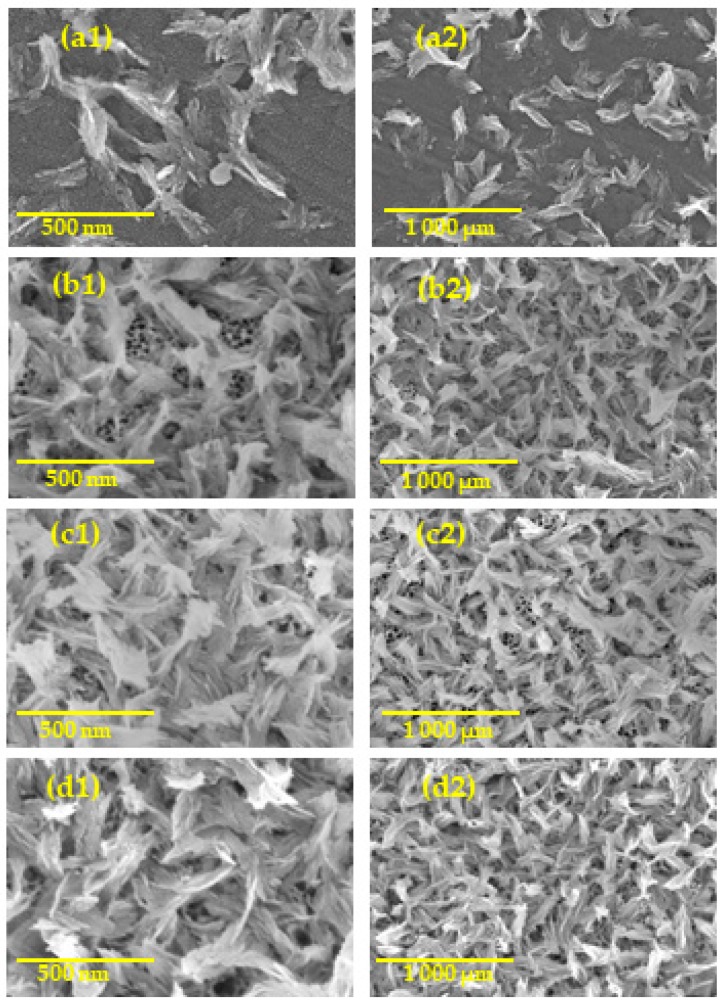
SEM images of (**a1**,**a2**)—Ti6Al4V/HA; (**b1**,**b2**)—Ti6Al4V/TNT5/HA, and (**c1**,**c2**)—Ti6Al4V/TNT12/HA, (**d1**,**d2**)—Ti6Al4V/TNT30/HA.

SEM images (Figure 4) revealed morphology differences of HA coatings deposited on the Ti6Al4V and Ti6Al4V/TNT substrates. The morphology of the hydroxyapatite layer significantly depends on the substrate type. Dispersed HA nanocrystals were formed on the surface of Ti6Al4V (Figure 4a), while on the TNT layers, the HA layers consisted of much more densely packed nanoplatelets. It should be noted that the HA nanoplatelet density significantly increases with the increase of the titania nanotube diameters (Figure 4b–d).

**Figure 5:**
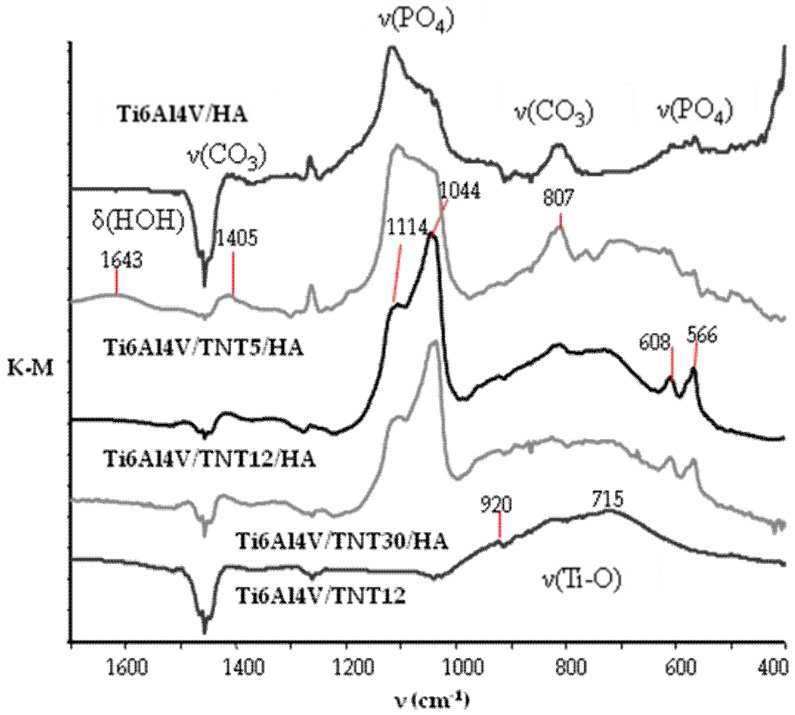
DRIFT spectra of studied Ti6Al4V/TNT, Ti6Al4V/HA, and Ti6Al4V/TNT/HA systems.

Figure 5 shows the diffuse reflectance infrared Fourier transformation spectra (DRIFT) of Ti6Al4V/TNT, Ti6Al4V/HA, and Ti6Al4V/TNT/HA samples. Absorption bands detected at ca. 1044, 1114 cm^−1^, and 566, 608 cm^−1^ were attributed to the vibrations of the phosphate groups of hydroxyapatite nanocrystals, while the bands at 807 and 1405 cm^−1^ were due to vibrations of CO_3_^2−^ groups [47]. Analysis of the HA and TNT/HA spectra draws attention to the lack of bands at 875–880 cm^−1^, which proves the absence of HPO_4_^2−^ groups. The wide band, which appears between 600 and 1000 cm^−1^, in the spectra of the Ti6Al4V/TNT/HA samples can be assigned to ν(Ti-O) modes of titania nanotubes. 

The hydrophilicity of the HA layers was estimated based on contact angle measurements of water droplets deposited on the sample surface (Table 2). The formation of dispersed HA nanocrystals on the surface of the Ti6Al4V sample increased the hydrophilicity so that the contact angle decreased from 108° to 57°. With the TNT/HA composites, the hydrophilicity increased further so that the values of the contact angles were in the range 32–16°, depending on the diameter of the titanium dioxide nanotubes. Analysis of changes in the surface free energy, estimated based on the Owens-Wendt method, which uses the values of contact angles for polar and dispersive liquid (water and diiodomethane, respectively), revealed only slight changes of its value for Ti6Al4V and Ti6Al4V/HA, and a clear increase for Ti6Al4V/TNT/HA systems from 65.8 mJ/m^2^ (TNT5/HA) up to 71.8 mJ/m^2^ (TNT30/HA).

### 3.2. The Mechanical Properties of the Studied Systems

**Figure 6:**
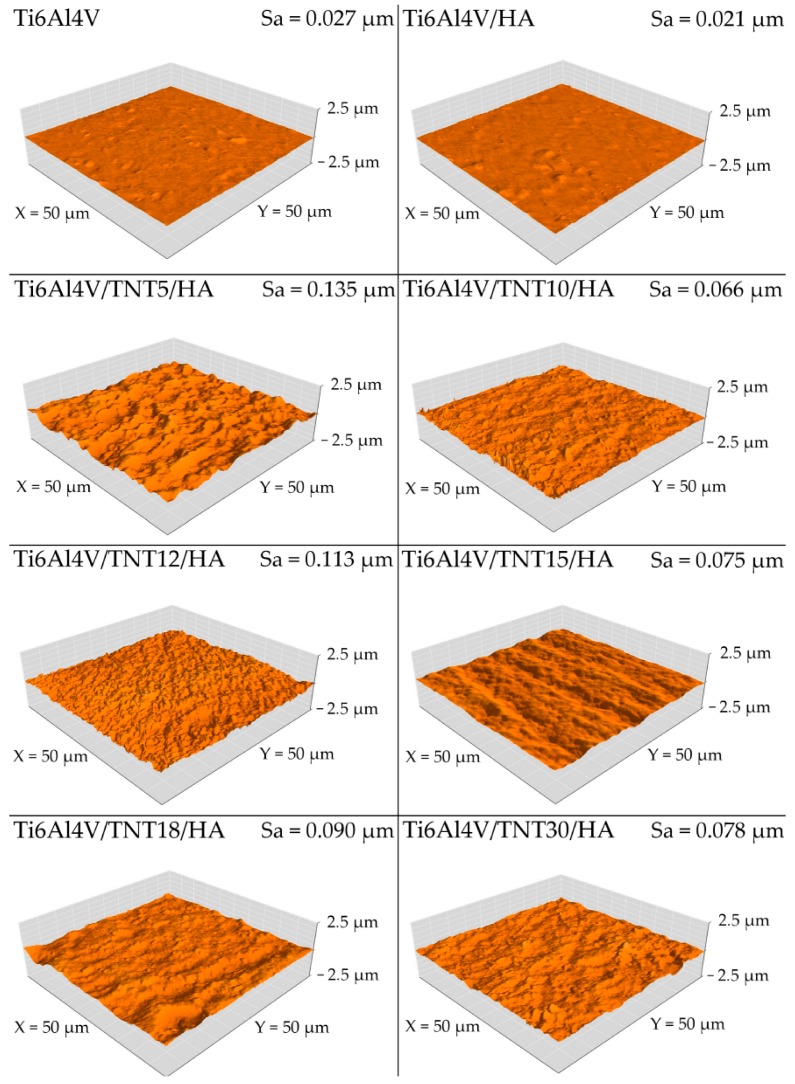
AFM surface topography and S_a_ parameters of the studied samples.

Deposition of hydroxyapatite on the surface of the titanium alloy practically does not change its surface roughness. By contrast, hydroxyapatite deposited on the nanotube layers caused a several-fold increase in roughness. However, there is no specific correlation between the S_a_ parameter and the diameter of the nanotubes (Table 3 and Figure 6). Similar relations occur for the hardness of the titanium alloy, Ti6Al4V alloy with the hydroxyapatite layer, and the anodized titanium alloy surface enriched with hydroxyapatite nanocrystals. The deposition of hydroxyapatite on the surface of the titanium alloy does not change its hardness, whereas the presence of TNT/HA composite reduces the hardness of the studied systems Ti6Al4V/TNT/HA Also, in this case, there is no correlation between the hardness and size of the nanotubes (Table 4). Scratch tests showed a decrease in the critical force that caused the loss of adhesion of the hydroxyapatite - TiO_2_ nanotube coatings deposited on the surface of the Ti6Al4V alloy (Table 5).

### 3.3. The Evaluation of Biointegration Properties of Ti6Al4V/TNT/HA

**Figure 7:**
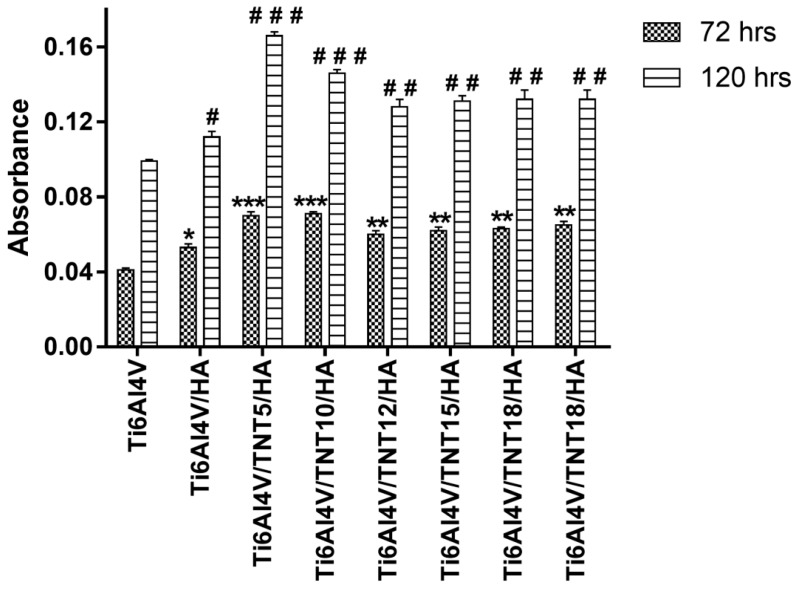
L929 murine fibroblasts proliferation (measured after 72 h and 120 h) on the surface of Ti6Al4V, Ti6Al4V/HA, and Ti6Al4V/TNT/HA, detected by the MTT assay. The absorbance values are expressed as means ± S.E.M of five independent experiments. Asterisks indicate significant differences in the level of cell proliferation observed after 72 h between the cells incubated with titanium alloy reference samples (Ti6Al4V) in comparison with the fibroblasts incubated on the surface of the tested samples (*** P < 0.001, ** P < 0.01, * P < 0.05). Hash marks denote significant differences in cell proliferation between Ti6Al4V reference samples and the tested specimens after 120 h (^# # #^ P < 0.001, ^# #^ P < 0.01, ^#^ P < 0.05).

The biointegration of the TiO_2_ nanocoatings enriched with a hydroxyapatite was evaluated based on the results of the MTT (3-(4,5-dimethylthiazol-2-yl)-2,5-diphenyltetrazolium bromide) assay and SEM images. The level of L929 murine fibroblasts’ proliferation measured after 72 h and 120 h is presented in Figure 7. It is worth noticing that with an increase of the incubation time, more L929 fibroblasts proliferated on all the tested materials (P < 0.001). Analysis of these data also revealed that the TNT/HA samples as well as the Ti6Al4V alloy foils covered with the hydroxyapatite (Ti6Al4V/HA) showed a higher level of cell proliferation than the Ti6Al4V reference sample after the both incubation times of 72 h and 120 h. This effect was the most noticeable for the Ti6Al4V/TNT5/HA and Ti6Al4V/TNT10/HA samples (P < 0.001).

**Figure 8:**
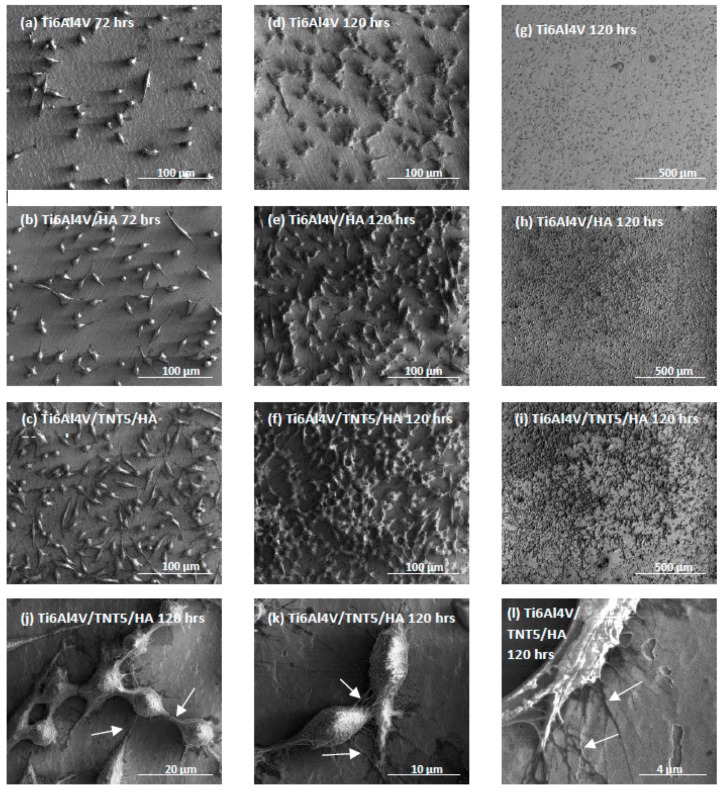
SEM micrographs showing the murine L929 fibroblasts proliferation (after 72 h and 120 h incubation times) on the surfaces of the Ti6Al4V reference alloy (**a**,**d**,**g**), Ti6Al4V alloy surface covered with the hydroxyapatite (Ti6Al4V/HA; **b**,**e**,**h**), and Ti6Al4V alloy covered with TiO_2_ and the hydroxyapatite (Ti6Al4V/TNT5/HA; **c**,**f**,**i**). Arrows indicate filopodia spread between the cells (**j**,**k**) or penetrating deep into the TNT5/HA nanocoatings after 120 h incubation time (**l**).

Comparative SEM images of L929 murine fibroblasts cultured on the selected nanocoatings covered with the hydroxyapatite are presented in Figure 8. Based on the results from the MTT assay, we have selected to show in Figure 8 those samples that induced the greatest and the least changes in cell proliferation compared to the Ti6Al4V reference samples (Ti6Al4V/TNT5/HA and Ti6Al4V/HA, respectively). SEM images confirm the results from the MTT assay and clearly indicate the high level of biointegration of the tested materials. As can be seen, the cells cultivated on the TNT5/HA nanocoatings effectively attached to the specimen surface and the number of L929 fibroblasts growing on the surface significantly increases over time. This observation is more noticeable for the Ti6Al4V/TNT5/HA specimens than for the Ti6Al4V/HA sample and the Ti6Al4V reference (compare micrographs a–c, d–f, and g–i in Figure 8). Importantly, the fibroblasts cultured for 120 h on the Ti6Al4V/TNT5/HA sample were crowded and formed networks due to the overgrowth of cells (Figure 8f), which clearly indicates that the tested nanocoatings stimulate cell proliferation. This observation was also confirmed by the elongated shape of the cells (e.g., see Figure 8c,k) and numerous filopodia, which attached the fibroblasts to the nanocoating surface by penetrating deep into the nanolayers (arrows in Figure 8l) or formed filopodia between the cells (arrows in Figure 8j,k).

### 3.4. The Evaluation of Antibacterial Properties of Ti6Al4V/TNT/HA

**Figure 9:**
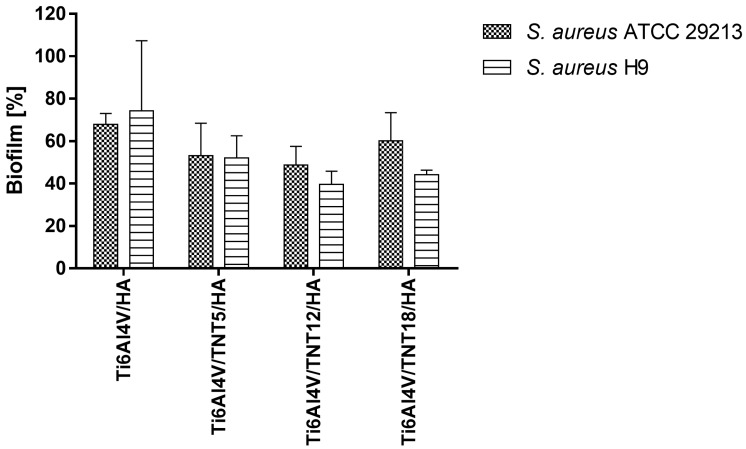
*S. aureus* ATCC 29213 and *S. aureus* H9 biofilms (24 h old) on the surfaces of Ti6Al4V/HA and Ti6Al4V/TNT/HA. The results are provided as the mean percentage ± standard deviation (SD) of staphylococcal biofilm compared to a control biofilm formed on the reference sample (Ti6Al4V), considered as 100%. Biofilm inhibition by all the tested samples in comparison to the reference Ti alloy was statistically significant (P < 0.05).

The samples of Ti6Al4V foil (unmodified control), titanium alloy foil coated with the hydroxyapatite (Ti6Al4V/HA), and titanium foil modified by both nanotubes and hydroxyapatite (Ti6Al4V/TNT/HA) were exposed for 24 h to staphylococci to assess their ability to inhibit bacterial colonization and biofilm formation. The presence of *S. aureus* ATCC 29213 reference strain and *S. aureus* H9 clinical strain biofilm on the surface of the samples was tested using a Live/Dead BacLight Bacterial Viability kit (L/D) and the results are presented in Figure 9 as a percentage of the live bacteria recovered from the biofilms on the tested coated samples in comparison to the biofilms formed on the unmodified Ti6Al4V control, considered as 100%.

All the tested modified titanium samples caused significant inhibition of staphylococcal colonization and biofilm formation in comparison to the reference Ti6Al4V sample, independently of the *S. aureus* strain used. The average percentage of biofilm inhibition, compared to the control biofilm developed on the unmodified titanium alloy considered as 100%, achieved the range from 32.1 ± 5.1% (P = 0.0008) to 51.3 ± 8.7% (P = 0.0008) and from 25.7 ± 33.0% (P = 0.0023) to 60.5 ± 6.2% (P = 0.0008) for *S. aureus* ATCC 29213 and *S. aureus* H9, respectively. The least potent in this respect was titanium alloy coated by the hydroxyapatite only (Ti6Al4V/HA) and additional nanostructural modification of the Ti6Al4V surface in the form of the nanotubes enriched with the hydroxyapatite (Ti6Al4V/TNT/HA) intensified their anti-biofilm activity. Among all the surfaces modified by both nanotubes and hydroxyapatite, the effect of Ti6Al4V/TNT18/HA against *S. aureus* ATCC 29213 was the weakest, reaching 39.9 ± 13.3% of biofilm inhibition. Both Ti6Al4V/TNT5/HA and Ti6Al4V/TNT12/HA showed higher antibacterial activity against this strain reaching, respectively, 47.0 ± 15.3 and 51.3 ± 8.7% of biofilm inhibition. Somewhat different, but even more clear, is the tendency visible for the *S. aureus* H9 strain. The effectivity of biofilm inhibition increased in the order of Ti6Al4V (0% of inhibition) < Ti6Al4V/HA (25.7% ± 33.0 of inhibition) < Ti6Al4V/TNT5/HA (48.1% ± 5.0 of inhibition <Ti6Al4V/TNT18/HA (55.8 ± 2.1 of inhibition) < Ti6Al4V/TNT12/HA (60.5 ± 5.2% of inhibition).

## 4. Discussion

Modification of the Ti6Al4V surface by biologically active coating materials, such as HA nanocrystals, TNT layers, and TNT/HA nanocomposites, is one of the most promising ways to improve the osseointegration processes [2,3,4,5,6,7,40]. Moreover, bioactive coatings can be also used for counteracting the formation of inflammation as well as for preventing the release of metal ions to the patient’s body during long-term use, e.g., in the construction of modern implants. According to the literature, TNT/HA coatings with good bioactivity have been produced by cathodic deposition [48] and pulse electrodeposition [2,3] of HA on the surface of TNT/Ti or TNT/Ti6Al4V substrates. In the both above-mentioned methods, the surface of the TNT coating was covered by densely packed nano- and micro-crystals of different shapes and sizes. 

In our work, we focused on the fabrication of HA coatings consisting of nanocrystals with a similar shape and size and good adhesion to the surface of titania nanotube coatings. Moreover, special attention was paid to the fact that the formed TNT/HA composite should exhibit beneficial properties of both TiO_2_ nanotubes and hydroxyapatite. Preservation of both constituents’ properties would not be possible if the hydroxyapatite layer completely covered the nanotubes layer as was in the case of both the above-mentioned methods. Therefore, we decided to exploit and modify the already reported method, which was applied to the production of HA coatings on the surface of unpolished Grade 2 titanium sheet [17,33]. This two-step method first deposits CaCO_3_ films on the titanium substrate by ALD, and then converts them to continuous hydroxyapatite coatings by wet treatment in a dilute phosphate solution [17]. Post-conversion annealing at 700 °C for 30 min, which was applied in that study, improved the adhesion test results by 0.8 MPa compared to non-annealed samples, but the difference was not statistically significant. In our work on the preparation of the TNT/HA composites, we had to consider that the TNT coatings cannot be heated to temperatures higher than 300 °C because that would cause the destruction of the nanotubular architecture [5]. To not cover the TiO_2_ nanotubes fully by the HA nanocrystals and simultaneously make them adhere well to the surface of the TNT layer, we decided to deposit significantly thinner HA films than in the previous studies. Based on our earlier experience, the number of CaCO_3_ ALD cycles was 100 in all our experiments. The results showed that the use of greater number of ALD cycles (i.e., 500 and 1000, which gave CaCO_3_ film of a thickness of 25 nm and 50 nm, respectively) resulted, after the conversion of CaCO_3_ into HA, in HA layers that were only weakly adherent to the TNT substrates. The HA layers deposited by 500 cycles on the surface of the Ti6Al4V/TNT substrates were delaminated and did not allow even for the proper performance of the biointegration MTT assay (Figure S1).

GIXRD and EDS confirmed that the HA films consisted of hydroxyapatite crystals, although the Ca/P ratios determined from the EDS spectra were lower than 1.67 (Table 1) [33,47]. The existence of bands characterized for CO_3_^2−^ in the IR spectra indicates the formation of carbonate substituted calcium deficient hydroxyapatite, with an overall formula Ca_10−x_(CO_3_)_x_(PO_4_)_6−x_(OH)_2−x_ (0 ≤ x ≤ 1), in which two types of carbonate substitutions are possible: (1) Direct substitution of OH^−^ with CO_3_^2−^ (a-type substitution CO_3_^2−^ ↔ 2OH^−^), and (2) CO_3_^2−^ substituting a tetrahedral PO_4_^3-^ group and charge compensation by calcium deficiency (B-type substitution) [47]. The lack of bands at 875–880 cm^−^^1^ in the DRIFT spectra proved that octacalcium phosphate (Ca_8_(HPO_4_)_2_(PO_4_)_4_·5H_2_O) or other species containing HPO_4_^2−^ ions of Ca/P = 1.3 or 1.5 were not formed (Figure 5). The fabrication of the Ti6l4V/TNT/HA structures without the calcination procedure led to the lack of calcium phosphate, Ca_3_(PO_4_)_2_, Ca/P = 1.5 [33,47].

**Figure 10:**
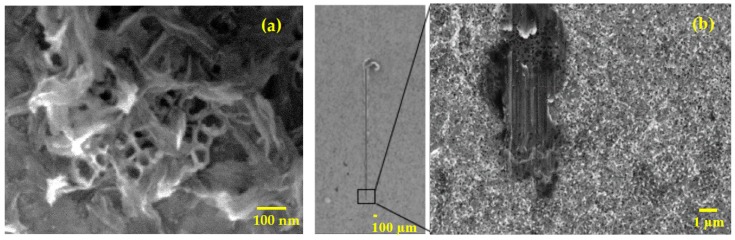
(**a**) HA nanocrystals on the surface of the nanotubes showing heterogeneous morphology with discontinuities. (**b**) The initial scratch trace after the scratch test for the sample Ti6Al4V/TNT15/HA.

SEM images of the surfaces of the Ti6Al4V/TNT5/HA–Ti6Al4V/TNT30/HA samples proved that the packing density of the HA nanocrystals on the surface of the TNT layers increases as the diameter of the nanotubes increases. Also, nanoindentation results highlight clear differences between the nanocrystalline HA coatings deposited on the surface of the TNT layers produced at different potentials. The HA coatings present heterogeneous morphology with discontinuities, which is a result of the distinct nanotubular architecture of the TNT layer on which the HA coating is formed (Figure 10a). Fabrication of TNT layers at 5 V (Ti6Al4V/TNT5) results in a significant decrease in the elastic modulus and nanohardness compared to the unmodified Ti6Al4V substrate. This effect is positive in the case of materials for long-term implants because it decreases the otherwise significant difference in mechanical properties between the implant and the bone and, consequently, a risk of the stress shielding effect [49]. The deposition of the HA coating on the surfaces of Ti6Al4V/TNT5 increased the H/E and H^3^/E^2^ ratios (H/E = wear resistance coefficient; H = hardness, E = Young’s modulus) compared to the uncoated Ti6Al4V/TNT system. For the Ti6Al4V/TNT15/HA system, the situation is reversed, i.e., the deposition of the hydroxyapatite reduced these ratios by 7% and 33%, respectively. The H/E and H^3^/E^2^ ratios influence the wear resistance of a material, either in bulk or in coating form. The first ratio characterizes the resistance of the material to elastic deformation, and the second ratio allows estimation of the ability of the material to dissipate energy upon plastic deformation during loading. The HA coating deposited on the Ti6Al4V/TNT5 improved the elasticity index and the resistance to plastic deformation, which suggests a better tribological performance of the HA coated surface in medical applications. The scratch test of the nanocomposites based on the HA-coated TNT surface did not give information about the adhesion of HA nanocrystals to TNT. The calculated critical forces were rather related to the loss of adhesion of the entire TNT/HA nanocoating to the Ti6Al4V substrate rather than to the loss of adhesion of HA to the nanotubes. Figure 10b presents the initial scratch trace, which shows the point of loss of adhesion of the TNT/HA coating for the Ti6Al4V/TNT15/HA sample. 

The mechanical properties of biomaterials used in the construction of implants should go hand in hand with their bioactivity. Therefore, studies on the ability of proliferation of fibroblast cells on the surface of the produced nanocomposites and on their potential antibacterial activity were carried out. Since mouse connective tissue fibroblasts, including L929 cells, are recommended as the standard model for biocompatibility testing [50], we chose this cell line to study the biointegration of the TiO_2_ nanocoatings enriched with the hydroxyapatite. Additionally, fibroblasts are considered to be the most common cells in connective tissue, one of the main components of peri-implant soft tissue, which is key to the formation of the peri-implant mucosal seal and helps to prevent epithelial ingrowth [51]. The results of the MTT assay indicated on the cell proliferation measured after 72 h and 120 h revealed the promising biocompatible properties of all the TNT coatings covered with the hydroxyapatite, with the highest level of proliferation observed for the TNT5/HA and TNT10/HA samples (Figure 7). Moreover, the results of the MTT assay correspond to the comparative SEM micrographs presented in Figure 8, which clearly showed the increase in the number of cells growing on the TNT5/HA coatings over time. This coating is characterized by the low surface energy (SFE = 65.84 mJ/m^2^) and the highest roughness (S_a_ = 0.135 µm) among the studied Ti6Al4V/TNT/HA samples. The surface roughness of biomaterials has a significant impact on the possibility of creating a proper connection between the implant and the bone. The influence of the implant surface roughness on the adhesion of cells and their proliferation has also been proven. A surface with a high roughness improves the adhesion of cells and tissues to the implant and provides improved primary stabilization between the implant and bone [52,53]. Analysis of SEM micrographs presented in Figure 8 also indicated that the L929 fibroblasts growing on the TNT5/HA coatings formed filopodia, which attached the cells to the nanotube arrays by penetrating deep into the nanolayers (white arrows in Figure 8l) and fibroblasts formed filopodia also among themselves (see white arrows in, e.g., Figure 8j,k). The ability of the cells to sense nanometer scale topographical features in their environment manifests itself, among others, in the formation of filopodia at the cell boundary, which enables the cells to spread over the nanocoatings [54]. Filopodia are cell protrusions consisting of a network of long parallel actin filaments, and therefore they play a fundamental role in cell attachment, migration, proliferation, and cell-cell interactions [55,56]. Our results correspond to the findings of other authors. Soares et al. demonstrated that the L929 fibroblasts’ adhesion and propagation is improved on the TiO_2_ nanotubes covered with a hydroxyapatite compared to an untreated titanium surface [57]. Similarly, hydroxyapatite deposited on TiO_2_ nanotubes showed higher cell density, more live cells, and better spreading of the mouse osteoblastic cell line, MC3T3-E1, as compared with an untreated titanium plate surface [58]. Taken together, these findings clearly demonstrate the biocompatible properties of the studied TNT/HA coatings, which are associated with the statement that a favorable cellular interaction with the biomaterials surface is critical for the long-term success of the implants [59]. 

Biofilm-associated infections of inserted and implanted biomaterials, such as intravascular catheters, prosthetic valves, orthopedic implants, vascular grafts, etc., remain a serious medical problem, which threaten the lives of patients. Therefore, the estimation of the antibacterial activity of the produced TNT/HA coatings, which could be applied in modifications of Ti6Al4V implant surfaces to limit potential infection development, was important for us. Earlier works revealed that the nanoarchitecture of the surface (e.g., TiO_2_ nanotubes, nanowires, nanofibers), metal ions/nanoparticles (mainly silver and copper), graphene oxide thin film, or covalently bounded antibiotics enhanced antimicrobial activity of titanium implants [5,7,60,61,62,63,64,65,66]. In our previous study, it was shown that TiO_2_ nanotubes formed on the Ti6Al4V alloy possessed in vitro anti-biofilm properties as tested on the *S. aureus* model [6]. The coatings obtained using low-potential anodic oxidation (3–5 V) were found to be the most effective, with the highest activity against staphylococci expressed by TNT5. Shen et al. demonstrated that carboxylated chitosan/silver-hydroxyapatite (CMCS/Ag-HA) hybrid microspheres exhibited in vitro good antimicrobial activity against *S. aureus* tested by the agar diffusion method. Although the choice of the method used was not the best one due to the lack of standardization in the reading of growth inhibition zones, the antimicrobial effect of the hybrid microspheres was noticeable and it was shown that CMCS/Ag-HA was more efficient than HA [67]. Similarly, in our research, double modified biomaterials exhibited stronger antimicrobial activity than the surface covered by the hydroxyapatite only, indicating a synergistic effect of both modifications. Herein, it should be remembered that the modifications of the biomaterials themselves are only one of the many elements influencing implant colonization by microorganisms in vivo. This has been well illustrated by Spengler et al., who used polished high-density pellets of hydroxyapatite as a model material for teeth to test *Streptococcus mutans* and nonpathogenic *Staphylococcus carnosus* adhesion in a human cavity, indicating the presence of saliva as a factor strongly determining the adhesion of *S. mutans*, but not *S. carnosus*, to HA [68]. Colonization of the implants by the microorganisms depends, inter alia, on the surface properties, microbial virulence factors, such as microbial surface components recognizing adhesive matrix molecules (MSCRAMMs), capsule and other adhesins, as well as the ambient conditions in the tissues, including the local immune response [64,69].

## 5. Conclusions

ALD was exploited to add hydroxyapatite on top of TiO_2_ nanotubes on a titanium alloy in an attempt to produce novel nanocomposites with optimized biocompatible, antibacterial, and mechanical properties for applications in medical implants. The results of our work revealed that the use of TiO_2_ nanotubes as a link system between the titanium alloy and a thin layer of hydroxyapatite nanocrystals seems to be an excellent solution to inhibit the delamination of the hydroxyapatite layer. Their nanoporous architecture led to a much stronger connection with hydroxyapatite than if the hydroxyapatite was deposited directly on the surface of the titanium alloy. However, one condition had to be fulfilled - the hydroxyapatite layer must be extremely thin. Such a thin layer can be provided by the method used in our experiment, i.e., the atomic layer deposition of CaCO_3_ films on the Ti6Al4V substrate followed by their conversion to hydroxyapatite nanocrystals by wet treatment in dilute phosphate solution. 

The results of our work indicate the promising properties of the Ti6Al4V/TNT5/HA as a bio-active nanocomposite. This system showed the best adhesion and proliferation of fibroblast cells, in comparison to the other studied nanocomposites, and simultaneously good antibacterial activity. Moreover, this system was characterized by high surface roughness, an appropriate elasticity index, and resistance to plastic deformation, which suggests optimal tribological performance of the surface for medical applications.

## Figures and Tables

**Table 1:** Ca/P ratios obtained from EDS measurements for the Ti6Al4V/HA and Ti6Al4V/TNT/HA samples.

Biomaterial Sample	Ca/P Ratio
Ti6Al4V/HA	1.65 ± 0.06
Ti6Al4V/TNT5/HA	1.62 ± 0.01
Ti6Al4V/TNT10/HA	1.59 ± 0.04
Ti6Al4V/TNT12/HA	1.47 ± 0.04
Ti6Al4V/TNT15/HA	1.54 ± 0.02
Ti6Al4V/TNT18/HA	1.45 ± 0.06
Ti6Al4V/TNT30/HA	1.54 ± 0.04

**Table 2:** Results of the contact angle and surface free energy (SFE) measurements; contact angles were measured three times using distilled water and diiodomethane and the SFE was determined using the Owens-Wendt method.

Sample	Average Contact Angle [°] ± Standard Deviation		SFE [mJ/m^2^]
	Measuring Liquid		
	Water	Diiodomethane	
Ti6Al4V	108.3 ± 0.1	37 ± 0.2	45.4 ± 0.1
Ti6Al4V/HA	57.4 ± 1.8	54.5 ± 2.6	44.6 ± 0.9
Ti6Al4V/TNT5/HA	32.2 ± 1.0	<10	65.8 ± 0.2
Ti6Al4V/TNT10/HA	23.3 ± 1.8	<10	69.6 ± 0.3
Ti6Al4V/TNT12/HA	22.2 ± 0.5	<10	70.5 ± 0.7
Ti6Al4V/TNT15/HA	18.9 ± 1.9	<10	71.6 ± 0.4
Ti6Al4V/TNT18/HA	18.2 ± 1.1	<10	71.3 ± 0.2
Ti6Al4V/TNT30/HA	16.5 ± 1.3	<10	71.8 ± 0.2

**Table 3:** Roughness of Ti6Al4V, Ti6Al4V/HA, and Ti6Al4V/TNT/HA composites.

Sample	S_a_ Parameter [µm]
Ti6Al4V	0.027
Ti6Al4V/HA	0.021
Ti6Al4V/TNT5/HA	0.135
Ti6Al4V/TNT10/HA	0.113
Ti6Al4V/TNT12/HA	0.066
Ti6Al4V/TNT15/HA	0.075
Ti6Al4V/TNT20/HA	0.090
Ti6Al4V/TNT30/HA	0.078

**Table 4:** Mechanical and nanoindentation properties of the studied samples.

Sample	Hardness [GPa]	Young’s Modulus [GPa]	Maximum Depth of Indentation [nm]
Ti6Al4V	16.29 ± 3.07	283.1 ± 30.9	161.1 ± 15.3
Ti6Al4V/HA	16.47 ± 2.38	293.5 ± 30.8	158.4 ± 9.7
Ti6Al4V/TNT5/HA	10.26 ± 4.08	256.3 ± 82.0	205.4 ± 46.5
Ti6Al4V/TNT10/HA	7.51 ± 1.40	126.1 ± 14.5	246.3 ± 20.1
Ti6Al4V/TNT12/HA	7.66 ± 1.70	248.3 ± 53.0	226.5 ± 32.6
Ti6Al4V/TNT15/HA	12.53 ± 3.30	291.6 ± 47.1	179.3 ± 23.5
Ti6Al4V/TNT20/HA	10.53 ± 2.59	160.8 ± 33.2	210.6 ± 31.5
Ti6Al4V/TNT30/HA	7.51 ± 1.48	239.9 ± 38.4	226.2 ± 22.4

**Table 5:** Results of nano scratch tests.

	Nano Scratch Test Properties	
Sample	Critical Friction [mN]	Critical Load [mN]
Ti6Al4V/HA	282.1 ± 52.8	229.0 ± 37.9
Ti6Al4V/TNT5/HA	165.6 ± 28.2	166.9 ± 21.7
Ti6Al4V/TNT10/HA	153.6 ± 39.6	139.2 ± 26.3
Ti6Al4V/TNT12/HA	124.0 ± 13.0	128.9 ± 13.0
Ti6Al4V/TNT15/HA	202.6 ± 45.0	175.5 ± 31.9
Ti6Al4V/TNT20/HA	149.3 ± 6.5	160.4 ± 13.4
Ti6Al4V/TNT30/HA	228.1 ± 54.7	204.1 ± 32.9

## Supplementary Materials

The following are available online at http://www.mdpi.com/2079-4991/9/1/123/s1, Figure S1: The formazan crystals in the culture medium (white arrows) formed during carrying out the MTT assay. Released formazan crystals indicate on the detachment of L929 cells from the surface of TiO_2_ nanotubes covered with a hydroxyapatite, obtained during ALD process applying 500 cycles.
