# Supplementary material for: Titania Nanotubes/Hydroxyapatite Nanocomposites Produced with the Use of the Atomic Layer Deposition Technique: Estimation of Bioactivity and Nanomechanical Properties
Source: Nanomaterials (Basel). 2019 Jan 19;9(1):123. doi: 10.3390/nano9010123 (PMC6359504; doi:10.3390/nano9010123)
Supplement: Supplementary file 1 [file nanomaterials-09-00123-s001.pdf]

Supplementary Materials

Titania Nanotubes/Hydroxyapatite Nanocomposites Produced with the use of the Atomic Layer Deposition Technique: Estimation of Bioactivity and Nanomechanical Properties

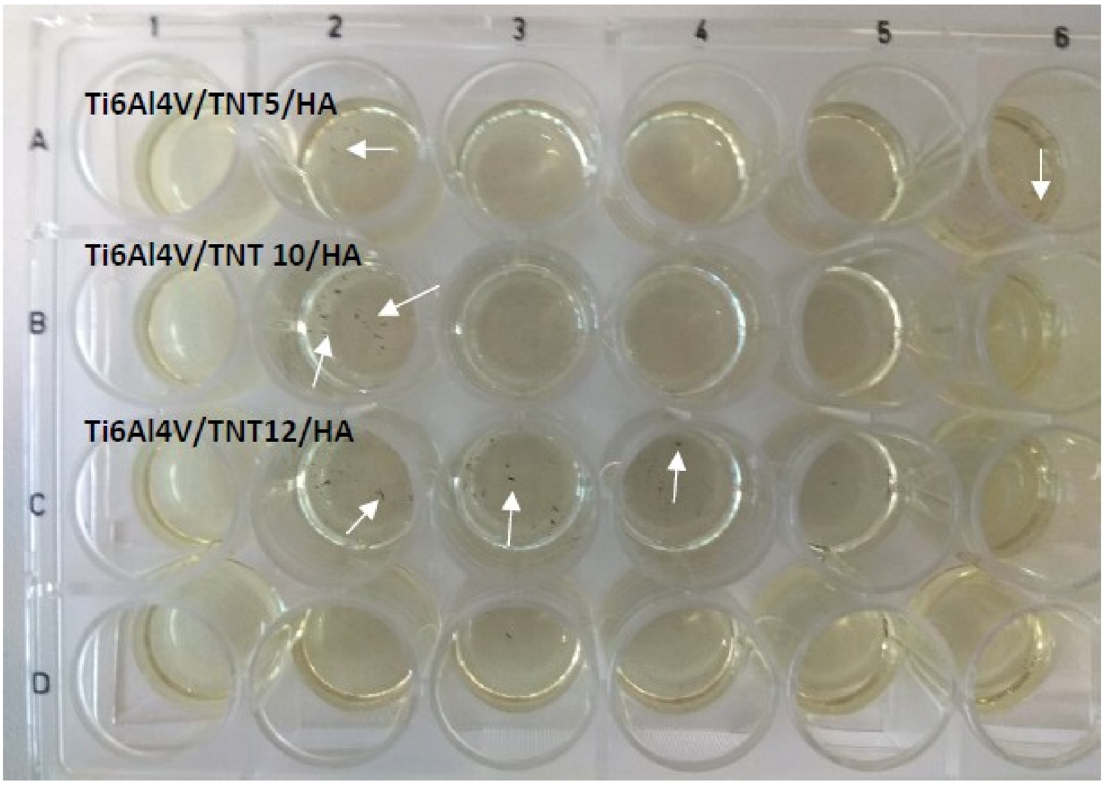

Figure S1. The formazan crystals in the culture medium (white arrows) formed during carrying out the MTT assay. Released formazan crystals indicate on the detachment of L929 cells from the surface of TiO_2 nanotubes covered with a hydroxyapatite, obtained during ALD process applying 500 cycles.
